# Membrane‐Associated Nucleobase‐Functionalized β‐Peptides (β‐PNAs) Affecting Membrane Support and Lipid Composition

**DOI:** 10.1002/cbic.202000172

**Published:** 2020-06-18

**Authors:** Geralin A. Höger, Markus Wiegand, Brigitte Worbs, Ulf Diederichsen

**Affiliations:** ^1^ Institut für Organische und Biomolekulare Chemie Georg-August-Universität Göttingen Tammannstraße 2 37077 Göttingen Germany

**Keywords:** aggregation, beta-peptides, membranes, peptide nucleic acids, peptide-peptide interactions

## Abstract

Protein‐membrane interactions are essential to maintain membrane integrity and control membrane morphology and composition. Cytoskeletal proteins in particular are known to interact to a high degree with lipid bilayers and to line the cytoplasmic side of the plasma membrane with an extensive network structure. In order to gain a better mechanistical understanding of the protein–membrane interplay and possible membrane signaling, we started to develop a model system based on β‐peptide nucleic acids (β‐PNAs). These β‐peptides are known to form stable hydrogen‐bonded aggregates due to their helical secondary structure, which serve to pre‐organize the attached nucleobases. After optimization of the β‐PNA solid‐phase peptide synthesis and validation of helix formation, the ability of the novel β‐PNAs to dimerize and interact with lipid bilayers was investigated by both fluorescence and circular dichroism spectroscopy. It was shown that duplex formation occurs rapidly and with high specificity and could also be detected on the surfaces of the lipid bilayers. Hereby, the potential of a β‐PNA‐based peptide system to mimic membrane‐associated protein networks could be demonstrated.

## Graphical abstract

**A model system** for membrane‐spanning protein networks – important for the stability of the membrane, its structure and transmembrane signal transmission – has been developed. The synthetically available system is based on β‐peptide nucleic acid (β‐PNA) helices functionalized with lipid membrane anchors and nucleobase recognition sequences. β‐PNA membrane interaction and aggregation are indicated by fluorescence and CD spectroscopy assays.

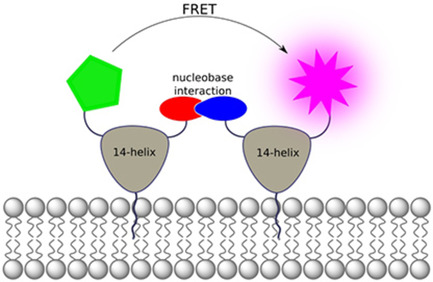

Protein‐membrane and protein‐protein interactions play a decisive role in numerous biological processes. Among other functions, these interactions are strongly involved in shaping and maintaining the structural integrity of the lipid bilayer as in the case of cytoskeletal protein networks. Spectrin forms complexes that line the plasma membrane and improve its stability and resistance to high mechanical stress. The fibrillar spectrin oligomers are anchored to the lipid bilayer via adaptor proteins, but it has also been shown that they only interact weakly directly with the membranes.^1^, ^2^ The cytoskeletal network formed by f‐actin is largely connected to the cell membranes via adaptor proteins and has been shown to alter lipid phase separation.^3^ In addition, these protein–membrane interactions have been reported to be associated with changes in local lipid composition and diffusion rates leading to the formation of membrane domains.^2^, ^3^ The hypothesis was derived that the cytoskeletal components in the immediate vicinity of the plasma membrane form compartments by their mesh size and fine‐tune membrane heterogeneity by cytoskeletal pinning.^2^, ^3^, ^4^, ^5^ In order to model these interactions in vitro, recombinant components of the eukaryotic cytoskeletal protein network were used.^2^, ^3^ Prokaryotic homologues or polymers were investigated to reduce complexity.^5^, ^6^

With the long‐term goal to gain further insights into the interdependence of cytoskeleton‐membrane interactions, a synthetic model system is proposed that has the potential to mimic these membrane‐interacting protein networks (Figure 1). It is assumed that conformationally stable peptide helices are anchored to the membrane surface by the introduction of amino acid lipid side chains. In addition, specific recognition units (nucleobases in this approach) are used to aggregate the helices on the membrane surface, with the possibility to shape the protein‐like scaffold as soon as the β‐peptide helical dimers are further interconnected by additional base pair recognition. By varying the type and amount of lipid anchors, the aggregation also has an influence on the lipid composition of the membrane, so that, for example, signaling effects by phase separation are possible and this β‐PNA network might be applied to the characterization of membrane kinetics and fluidity.

**Figure 1:**
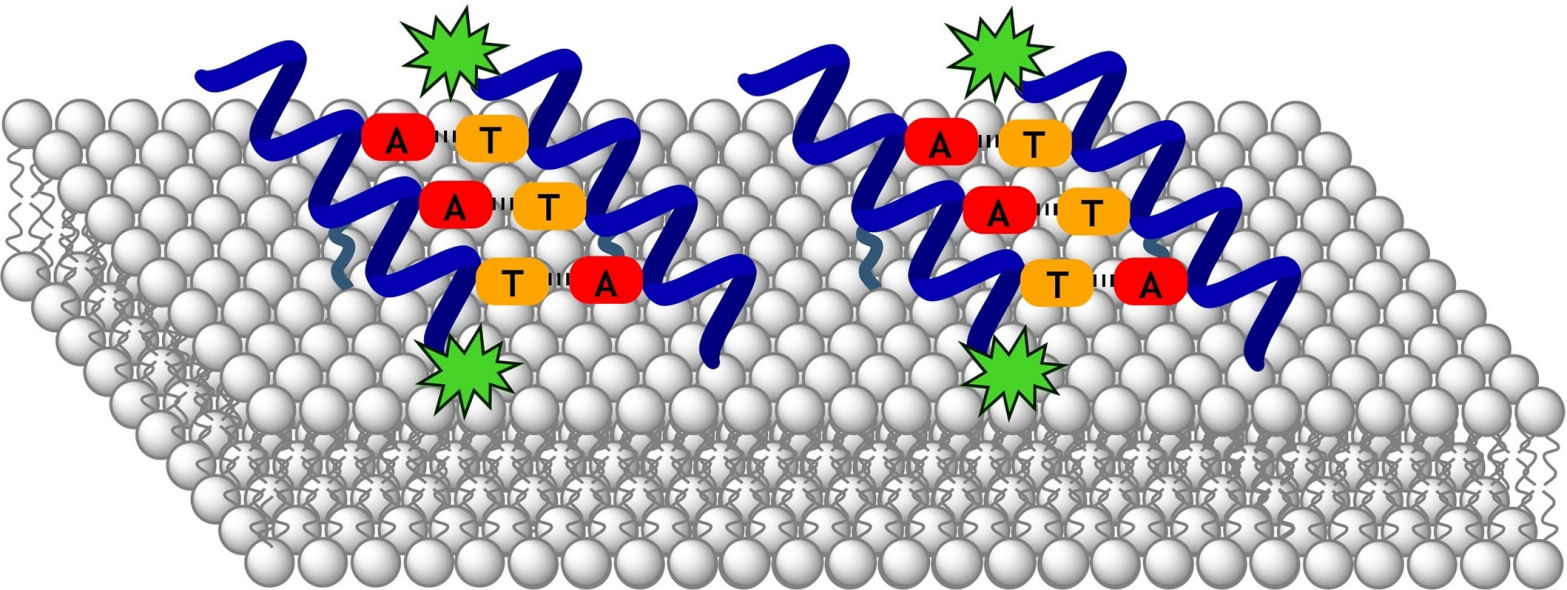
Model of a membrane‐interacting protein network consisting of peptide helices anchored on the membrane surface by lipid side chains (dark green) and nucleobase recognition (red and orange). Fluorophores (green) are attached for analytical reasons.

Similar to its biological counterpart, the artificial system should form fibrillar aggregates. In our approach, β‐peptides were chosen as scaffolds because they form conformationally stable and rigid helix structures even at short sequence lengths.^7^, ^8^, ^9^ In particular, the topology of a 14‐helix is suitable as building block because it contains exactly three amino acids per helix turn, therefore offers three different functional helix surfaces and has a pitch of about 5 Å.^8^, ^9^, ^10^, ^11^, ^12^ Due to their highly stable and regular structure and arrangement of side chains in three faces and the freedom regarding side chain functionalization, β‐peptides offer a suitable platform for the required molecular architecture.

Peptide aggregation based on the molecular recognition of nucleobases has already been described using d‐β‐homo‐alanine nucleobase building blocks on a helix surface leading to the formation of stable antiparallel duplexes.^13^, ^14^ The specific interaction of these β‐peptide nucleic acids (β‐PNA) has been used to study SNARE protein analogue‐mediated membrane fusion.^15^ In addition, higher aggregates of β‐peptides containing a bifacial nucleobase modification were investigated, which serve as a good starting point for aggregation networks on membrane surfaces.^16^ β‐PNA recognition based on AT sequences allow for specific base pairing, however, sequences with high CG content also interact nonspecifically, so that in this study fine‐tuning of the sequence design is required.^17^

As a starting point for the investigation of aggregation in combination with membrane interaction, a helix surface was functionalized with three nucleobases (Nb) by incorporating d‐β‐homoalanine nucleobase building blocks into the sequence. To test the antiparallel interaction of dimers (Figure 2a), sequences were designed by starting with the weakest interaction motif AAT−ATT and gradually increasing the GC content until only one A−T base pair remained to ensure sequence specificity (Figure 2b). The second face of the helix was modified with a myristyl (C_14_) or methyl alcohol by esterification of a d‐β‐aspartic acid side chain to provide a hydrophobic group that interacts with the lipid bilayer.^18^ Additionally, *trans*‐(1*R*,2*R*)‐2‐aminocyclohexane carboxylic acid (ACHC) was integrated to stabilize the helical conformation.^9^ To prevent incorporation of β‐peptides as transmembrane helices, the helical surface hydrophilicity was increased by the use of d‐β‐homo‐lysine.

**Figure 2:**
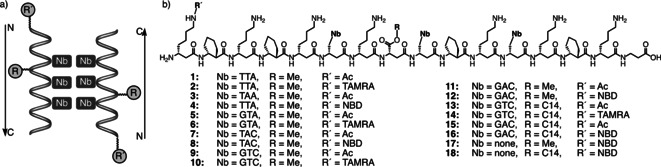
a) Model of antiparallel dimer formation of nucleobase (Nb)‐functionalized β‐peptides. b) Amino acid sequence of synthesized β‐peptides with modifications at the N‐terminal homolysine and aspartic acid side chains.

β‐Peptides **1**–**18** were synthesized by manual microwave‐assisted solid‐phase peptide synthesis (SPPS) with HOAt and HATU as activators at 65 °C (see the Supporting Information for experimental details). In addition to shorter coupling times, the building blocks were used with reduced stoichiometry making the overall synthesis more efficient than methods described previously. For FRET the β‐peptides were either labeled with 7‐nitrobenz‐2‐oxa‐1,3‐diazol‐4‐yl (NBD) or 5(6)‐carboxytetramethylrhodamine (TAMRA) or they were used as reference in their acetylated form. The β‐peptides were purified by high‐performance liquid chromatography and the structural integrity was proven by mass spectrometry. In total, β‐peptides with six different nucleobase sequences (**1**–**16**) and two control β‐peptides lacking nucleobases were prepared (**17**–**18**, Figure 2b).

Circular dichroism (CD) spectroscopy was performed to confirm the 14‐helix conformation (Figure S1). For all β‐peptides, the characteristic CD signals were found, indicating the formation of a 14‐helix with a maximum between 210 and 215 nm, a zero crossing between 200 and 204 nm and a minimum at about 195 nm.^19^ In addition, a band between 260 and 300 nm was observed due to the conformational orientation of the nucleobases.^16^, ^19^

The dimerization of β‐peptides by nucleobase recognition in aqueous solution was analyzed by fluorescence spectroscopy, whereby the β‐PNA oligomers were labeled with NBD or TAMRA. The fluorescence intensity of NBD was measured at 530 nm and 10 °C as a function of the different TAMRA‐labeled β‐peptide concentrations. The total peptide concentration was kept constant by addition of the corresponding acetylated β‐PNA. In addition to the complementary combinations **1**/**2**+**4**, **5**/**6**+**8** and **9**/**10**+**12**, all possible mismatch combinations **1**/**2**+**8**, **1**/**2**+**12**, **5**/**6**+**4**, **5**/**6**+**12**, **9**/**10**+**4** and **9**/**10**+**8** were measured to gain insight into the sequence specificity of the system.

All TAMRA‐labeled and acetylated β‐peptides were also combined with control peptide **17**, which lacked nucleobases (see the Supporting Information for fluorescence data). In the complementary combinations of **1**/**2**+**4** and **5**/**6**+**8**, a slight change in NBD fluorescence emission was observed (Figure 3a), while **9**/**10**+**12** showed a significant FRET, which can be clearly distinguished from the control peptide **9**/**10**+**17** without detectable FRET (Figure 3b). Furthermore, the mismatch combinations **9**/**10**+**4** and **9**/**10**+**8** showed the same results as the negative control, thus indicating high sequence specificity. When the measurements were repeated at 20 °C, the FRET was less pronounced for **9**/**10**+**12** and was no longer observable for **5**/**6**+**8** or **1**/**2**+**4** (Figure S5). These results show that the β‐PNA interaction is highly sequence specific and can be modulated by sequence and temperature.

**Figure 3:**
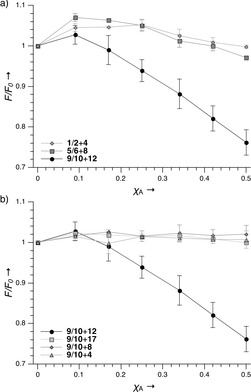
Relative changes in NBD fluorescence intensity (*F/F*_0_) at 530 nm as a function of increasing molar fraction of the complementary TAMRA‐labeled β‐PNA strands (*Χ*_A_). a) The results of all matching combinations and b) of **9**/**10** with all possible combinations are shown. Measurements were performed at 10 °C in 10 mM Tris**⋅**HCl buffer at pH 7.5 (number of measurements *n*=3).

The CD measurements were performed at 20 °C to validate the FRET results (Figure 4). The CD spectra of **9**+**11** showed an increased signal intensity in all absorption bands compared to the individual spectra (Figure 4a), indicating an interaction between the β‐peptides. In contrast, the CD spectra of **5**+**7** and **1**+**3** showed no significant increase in CD signal (Figure S6). Interestingly, it could also be shown that no annealing is necessary for the peptide interaction, as the CD spectra of **9**+**11** with and without pre‐incubation at 80 °C are identical. The CD melting curves for **9**+**11** were performed at the absorption maximum of the nucleobases at 273 nm, and a sigmoidal shape was recorded for **9**+**11** (Figure 4b), indicating a cooperative binding mode.^13^, ^20^ Oligomers **5**+**7** and **1**+**3** showed a less pronounced temperature‐dependent change in molar ellipticity, indicating that in these cases the interaction occurs only at low temperatures. Melting temperatures of 40 and 15 °C were determined for **9**+**11** and **5**+**7**, respectively.

**Figure 4:**
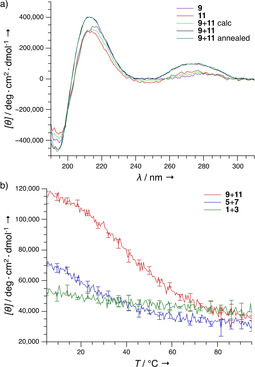
a) CD spectra of **9** and **11** at 20 °C measured separately, and as equimolar mixture (with and without annealing from 80 °C); the calculated average is indicated. b) Temperature dependence of the CD spectra at 273 nm of the combinations **9**+**11**, **5**+**7** and **1**+**3**. Measurements were performed in 10 mM Tris**⋅**HCl puffer at pH 7.5 (*n*=3).

Based on these promising results in solution, the peptide interaction between β‐peptides containing the GTC and GAC nucleobase motifs was investigated with lipid bilayer associated peptides. The duplex stability of the peptides **9**+**11** is also suitable for physiological conditions, especially since higher stability can be expected once a hydrogen‐bond network is based on β‐peptides with two flanks modified with recognition units and when recognition is supported by the membrane anchorage. Therefore, anchoring of β‐PNA helices in the membrane was ensured by adding the NBD‐labeled peptides **17** (Me) and **18** (C_14_) to large unilamellar vesicles (LUVs) containing DOPC and lipid head group‐bound lissamine‐rhodamine B. Only for **18** significant FRET was detected, indicating that the myristyl moiety allows spontaneous and autonomous interaction with the membrane (Figure S7). FRET between TAMRA and NBD with labeled **14** and **16** was measured in 1,2‐dimyristoyl‐*sn*‐glycero‐3‐phosphocholine/1,2‐diheptanoyl‐*sn*‐glycero‐3‐phosphocholine (DMPC/DHPC) vesicle solutions, as the DOPC LUVs would aggregate upon addition of the β‐peptides. Fluorescence emission spectra showed that after the addition of **14** the NBD fluorescence emission of **16** was reduced and TAMRA fluorescence emission was observed. This showed close proximity between **14** and **16** and thus an interaction between these peptides (Figure 5a). Time‐resolved FRET assays were performed, recording the NBD emission of **16** at 530 nm to assess how fast the interactions occur. As shown in Figure 5b, the addition of **14** led to a rapid decay of NBD fluorescence within 60 s. When repeating the measurements with the control peptide (**18**+**14**), no FRET was observed (Figure S8b), indicating that the β‐PNA interaction is specific and efficient. Measurements with and without vesicles showed amplification of NBD fluorescence emission in the presence of lipid bilayers (Figure S8). Since the fluorescence emission of NBD is increased in hydrophobic environment,^21^ these measurements indicate rapid binding of the myristyl‐modified β‐peptides.

**Figure 5:**
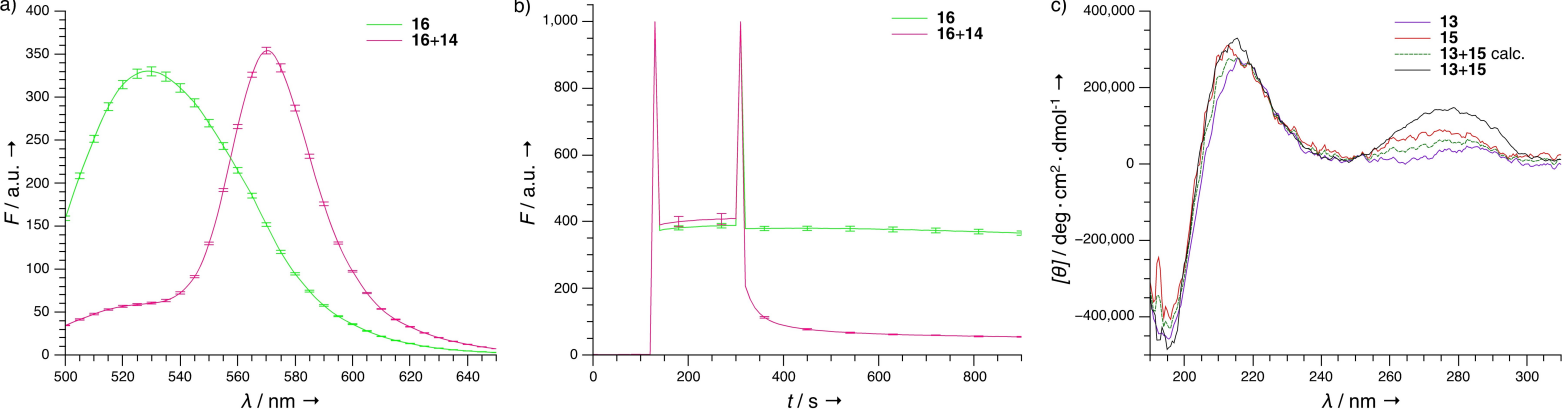
β‐PNA interaction on the surface of DMPC/DHPC bilayers. a) Fluorescence emission spectra of **16** with and without equimolar addition of **14** as well as b) time‐resolved NBD fluorescence emission measurements at 530 nm of the same peptide combination. c) CD spectra of **13** and **15** measured separately and as an equimolar mixture; the calculated average is also indicated. Measurements were performed at 20 °C in 5 mM Tris**⋅**HCl buffer at pH 7.5 with DMPC/DHPC vesicles (*q*=2, *n*=3).

In order to validate the results obtained with FRET, CD measurements with **13**+**15** in DMPC/DHPC bilayers were performed. The CD spectrum of **13**+**15** showed a significantly higher CD signal in the nucleobase absorption band compared to the single spectra or the calculated average (Figure 5c), which supports the assumption that β‐PNA dimerization also occurs on lipid bilayers. In addition, CD spectra for **13** and **11** were recorded to investigate whether peptide interaction also occurs when only one β‐peptide is anchored in the lipid bilayer (Figure S9b). Although only a small increase in CD signal was observed in the nucleobase absorption region, the difference obtained for **13**+**11** is significant compared to the calculated average and could be caused by β‐PNA/β‐PNA interaction. The results show that the myristyl‐modified β‐peptides are not immersed in the lipid bilayer as the absolute minimum and maximum values correspond to those of their soluble counterparts. In case of membrane insertion, the CD signal would have been shifted to shorter wavelengths.^22^ In further experiments, the β‐peptide membrane interaction will be supported by atomic force microscopy (AFM), which provides more information about the distance between β‐peptides and the lipid bilayer.

In conclusion, a model system for membrane scaffolding proteins based on β‐peptide helices was established, which contains membrane anchoring lipids on the one hand and nucleobase recognition units on the other hand. Based on the conformational rigidity of a β‐peptide 14‐helix, the three sides of the helix were specifically functionalized, and the interaction with the membrane and the aggregation of the helices was followed by fluorescence spectroscopic methods. Evidence was found for β‐PNA helices anchored by myristyl side chains on the membrane surface. Furthermore, sequence‐specific dimerization of β‐PNAs in solution and on the membrane surface was detected. This provides conceptual proof of principle for the use of these β‐PNA oligomers as model systems for membrane scaffold proteins. Further studies will aim to extend the β‐PNA recognition system on two sides of the helix to allow higher aggregates on lipid bilayer surfaces. Regulatory effects will be investigated that result either from the defined shape of the protein‐like β‐PNA scaffold on the membrane surface or from the aggregation‐dependent tuning of the membrane lipid composition. Further application of this β‐PNA network could result from the potential of encapsulation and stabilization of vesicles with regard to new drug delivery technologies.^23^

## Conflict of interest

The authors declare no conflict of interest.

## Supporting information

As a service to our authors and readers, this journal provides supporting information supplied by the authors. Such materials are peer reviewed and may be re‐organized for online delivery, but are not copy‐edited or typeset. Technical support issues arising from supporting information (other than missing files) should be addressed to the authors.

Supplementary
