# Supplementary material for: Membrane‐Associated Nucleobase‐Functionalized β‐Peptides (β‐PNAs) Affecting Membrane Support and Lipid Composition
Source: Chembiochem. 2020 Jun 18;21(18):2599–603. doi: 10.1002/cbic.202000172 (PMC7540700; doi:10.1002/cbic.202000172)
Supplement: Supplementary file 1 — Supplementary [file CBIC-21-2599-s001.pdf]

ChemBioChem

Supporting Information

Membrane-Associated Nucleobase-Functionalized β-Peptides (β-PNAs) Affecting Membrane Support and Lipid Composition

Geralin A. Höger, Markus Wiegand, Brigitte Worbs, and Ulf Diederichsen*

Supporting Information

Contents

Contents 1
Material and Methods 2
Solvents & Reagents 2
Reactions 2
HPLC 2
Mass Spectroscopy 2
UV-Spectroscopy 2
CD-Spectroscopy 2
Fluorescence Spectroscopy 3
Preparation of Large Unilamellar Vesicles[2] 3
Preparation of Bicelles[3] 4
Synthesis of Building Blocks 4
Synthesis of D-Nucleo-β^3-amino acids 4
Synthesis of D-β^3-amino acids 4
Synthesis of the β-Peptides 1-18 4
Analytical Data of the β-Peptides 5
CD Spectra 7
Concentration-dependent Fluorescence Spectra of the β-Peptides 7
Temperature-dependent CD Spectra of the β-Peptides 10
FRET Assay between β-Peptides 17, 18 and DOPC-LUVs 10
FRET Assay between β-Peptides 16, 18 and 14 on Lipid Bilayers 11
CD Spectra of the β-Peptide Interaction on Lipid Bilayers 11
Characterization of LUVs and bicelles by DLS 12
References 12

Material and Methods

Solvents & Reagents

Solvents were purchased and used in analysis grade. Solvents for HPLC were HPLC grade. Ultra-pure water was obtained from an Arium Mini water purification device by Sartorius (Göttingen, Germany). Commercially available reagents were of the highest grade available and used as supplied. Resins were obtained from IRIS Biotech GmbH (Marktredwitz, Germany). Amino acids were supplied by Novabiochem (Darmstadt,Germany), GL Biochem (Shanghai, China), PolyPeptide Group (Strasbourg, France), Fluorochem (Hadfield, UK) and Oxchem (Wood Dale, USA). Coupling reagents were obtained from Iris Biotech (Marktredwitz, Germany), GL Biochem (Shanghai, China), Fluorochem (Hadfield, UK) and Roth (Karlsruhe, Germany). Lipids were supplied by Avanti Polar Lipids, Inc. (Alabaster, USA). All other chemicals were acquired from Acros Organics (Geel, Belgium), Alfa Aesar (Karlsruhe, Germany), Applichem (Darmstadt, Germany), Fisher Scientific (Schwerte, Germany), Fluka (Taufkirchen, Germany), Fluorochem (Hadfield, UK), Glentham Life Science (Corsham, UK), Grüssing (Filsum, Germany), Iris Biotech (Marktredwitz, Germany), Merck (Darmstadt, Germany), Roth (Karlsruhe, Germany), and VWR Chemicals (Fontenay-sous-Bois, France).

Reactions

Air or moisture sensitive reactions were carried out under inert argon or nitrogen atmosphere and in dry solvents. All utilized glassware was heated under reduced pressure for drying beforehand and flushed with inert gas prior to use.

HPLC

RP-HPLC for analysis and purification of samples was performed using an HPLC system from JASCO (Tokyo, Japan), consisting of a diode array MD-2010plus, degasser DG-2080-53 and two PU-2080plus pumps. Optionally, a fraction collector CHF122SC from Advantec (Milpitas, USA) was coupled to the HPLC system. As solvents H_2O + 0.1 % (solvent A) and acetonitrile + 0.1 % TFA (solvent B) were used with s flow rate of 1 mL/min for analytical runs and 3 mL/min for semipreperative runs. RP-C18-ec Nucleodur columns from Macherey-Nagel (Düren, Germany) were used (particle size: 5 µm, pore size: 100 Å, column length: 250 mm, column diameter: 4.6 mm (analytical), 10 mm (semipreparative)). Peptides were dissolved in H2O/MeCN or HFIP and filtered before HPLC and UV-detection was performed at 215 nm, 245 nm and 280 nm for non-labelled β-peptides. For labeled β-peptides, UV-absorption was recorded at 464 nm for NBD and 540 nm for TAMRA instead of 280 nm.

Mass Spectroscopy

Electrospray ionization (ESI-MS) and high-resolution ESI (HR-MS) spectra were recorded with a maXis or MicroTOF spectrometer from Bruker Daltonik GmbH (Bremen, Germany). The values are given in m/z.

UV-Spectroscopy

Determination of β-peptide concentrations was conducted using a nanodrop ND-2000c spectrophotometer from Thermo Scientific (Munich, Germany) either by using the implemented pedestal method or with a Quartz SUPRASIL QS cuvette of 1.0 cm path length from Hellma Analytics (Müllheim, Germany). Concentrations were calculated from the absorbance using the Lambert-Beer's law.

CD-Spectroscopy

CD spectroscopy was performed with a J-1500 CD spectrometer from Jasco (Tokyo, Japan) and an F250 recirculation cooler from Julabo (Seelbach, Germany). Measurements were performed with a Quartz SUPRASIL QS cuvette with a 0.1 cm path length from Hellma Analytics (Müllheim, Germany). During measurements, the device and sample cell were flushed with nitrogen. The spectra were recorded in a wavelength range from 180 nm to 350 nm with a bandwidth of 1.0 nm, a data pitch of 0.5 nm, a response time of 1.0 s, a scanning speed of 100 nm/min and a CD scale of 200 meg/1.0 dOD in 'continuous mode'. Measurements were performed with the indicated temperatures and in the indicated solvents. An accumulation of five spectra was recorded per sample and background-corrected

against pure solvent without β-peptide. Afterwards, the spectra were expressed as molar ellipticity *[θ]* (deg × cm2 × mol−1) according to GREENFIELD.[1]

For temperature-dependent CD measurements, Samples were heated with a heating rate of 1 °C/min from 5 °C to 95 °C. Data points during heating and cooling cycles were recorded with a sampling rate of 0.5 °C, 1 s wait time, D.I.T. of 2 s, CD scale of 200 mdeg/1.0 dOD and bandwidth of 1.0 nm at 273 nm wavelength. Melting temperatures for the dimerizing β-peptides were determined by the peak maximum of the first derivation of the fitted melting curves. Additionally, spectra scans were performed during heating at 5 °C, 20 °C, 40 °C, 60 °C, 80 °C and 95 °C in a wavelength range from 180 nm to 320 nm with a bandwidth of 1.0 nm, a scanning speed of 100 nm/min, a data pitch of 0.5 nm, a D.I.T. of 2 s and CD scale of 200 meg/1.0 dOD in ‘continuous mode’. An average of five spectra was recorded per sample and background-corrected against pure solvent without β-peptides. Afterwards, the spectra were expressed as molar ellipticity as described before.

Fluorescence Spectroscopy

Concentration-dependent fluorescence spectra and FRET assays between NBD-labeled and TAMRA-labeled β-peptides were measured with an FP-6200 spectrometer from Jasco (Seelbach, Germany) with an ETC-272T temperature controller from Jasco (Seelbach, Germany) and a WKL26 recirculation cooler from HAAKE (Karlsruhe, Germany). Measurements were conducted at the indicated temperatures as well as solvents and with a Quartz SUPRASIL QS fluorescence cuvette of 1.0 cm path length from Hellma Analytics (Müllheim, Germany). While the TAMRA-labeled β-peptide concentration was varied with the mole fraction X_A varying from 0.0 to 0.5, the NBD-labeled β-peptide concentration with 4 µM as well as the total β-peptide concentration with 8 µM was kept constant by addition of the corresponding acetylated β-peptide. Emission spectra were recorded in a wavelength range from 470 nm to 650 nm with excitation at 460 nm, bandwidth of 5 nm, response set to ‘fast’, scanning speed of 125 nm/min, data pitch of 1.0 nm and sensitivity set to ‘medium’.

Time-resolved measurements were started with Tris-HCl buffer (5 mM, pH 7.5) which contained either 1 mM DMPC/DHPC (q=2) or 0.3 mM DHPC or no lipids at all in a cuvette with a stirrer at 20 °C. After 120 s the NBD-labeled β-peptide was added to yield a concentration of 0.5 µM. The NBD fluorescence emission was recorded for 180 s before the TAMRA-labeled β-peptide (0.5 µM) or buffer was added and the fluorescence emission was further recorded for 600 s. Excitation was set to 460 nm and the fluorescence intensity was detected at 530 nm with a bandwidth of 5 nm, a data pitch of 10 s, response set to ‘fast’, measure time set to 900 s and sensitivity set to ‘high’.

FRET assays to investigate membrane interaction of the β-peptides were performed with a Clariostar plate reader from BMG Labtech (Ortenberg, Germany) at room temperature in a black pp 96-well F-bottom plate from Greiner Bio-One (Kremsmünster, Austria) with a total sample volume of 200 µL. NBD-labelled β-peptides and Rhodamine-labelled LUV (0.75 %) were mixed right before measurement at a P/L ratio of 1:150 in Tris-HCl buffer (10 mM, pH 7.5). After focal and gain adjustment, emission spectra were recorded with an excitation wavelength of 460 nm in a range of 480–660 nm while the bandwidth was set to 10 nm.

Preparation of Large Unilamellar Vesicles[2]

LUVs were prepared according to the following protocol modified from MACDONALDS *et. al.*:[2]
The required lipids were dissolved in $CHCl_3$ on ice and the required amount was transferred to small glass test tubes. The solvent was removed with a nitrogen stream yielding a clear lipid film on the inner test tube walls. The lipid films were dried overnight under reduced pressure at 50 °C. Then, the lipid films were rehydrated in up to 1 ml filtrated buffer by 1 h incubation either at 40 °C for DMPC lipids or at rt for DOPC lipids. Subsequent vortexing for 30 s followed by 5 min incubation in three cycles yielded a milky MLV suspensions. The suspensions were loaded into a LiposoFast extruder with gas-tight syringes from Avestin (Ottawa, Canada). The vesicle suspensions were extruded 31 times through polycarbonate membranes with a pore size of 100 nm to yield a clear LUV suspension.

Preparation of Bicelles[3]

DMPC/DHPC bicelles were prepared according to the following protocol modified from DeAngelis *et. al.*:[3]
The required amounts of DMPC dissolved in $CHCl_3$ were transferred to small glass test tubes. The solvent was removed with a nitrogen stream resulting in clear lipid films on the inner test tube walls. The lipid films were dried overnight at 50 °C and under reduced pressure. The lipid films were dissolved in 20 mM DHPC in Tris-HCl buffer (5 mM, pH 7.5) to yield a DMPC concentration of 40 mM and a $q_{DMPC/DHPC}$ of 2. Subsequent vortexing for 30 s, incubation on ice for 5 min and heating to 42 °C for 10 min in three cycles yielded a clear bicelle solution. The bicelle solution was stored on ice and diluted shortly before measurements to keep it stable.

Synthesis of Building Blocks

Synthesis of D-Nucleo-β³-amino acids

Synthesis of Boc-D-β³-ʰala(G)-OH, Boc-D-β³-ʰala(C^Z)-OH, Boc-D-β³-ʰala(A)-OH and Boc-D-β³-ʰala(T)-OH were performed as described previously.[4]

Synthesis of D-β³-amino acids

Boc-D-β³-ʰlys(Cbz-Cl)-OH and Boc-D-β³-ʰlys(Fmoc)-OH were synthesized from commercially available Boc-D-lys(Cbz-Cl)-OH and Boc-D-lys(Fmoc)-OH by Arndt-Eistert homologisation as described previously.[5]

Synthesis of the β-Peptides 1-18

The required β-peptides were synthesized by manual microwave-assisted SPPS. Synthesis was performed in a 20 µmol scale in a PE-frit equipped BD discardit II syringe. For the coupling steps a discover microwave (MW) reaction cavity from CEM (Kamp-Lintfort, Germany) was used. First, Boc-β-Ala-PAM resin was swollen in DMF overnight. Cleavage of the Boc-protecting group was performed using TFA with 5 % m-cresol (v/v, 2 × 2.0 mL, 1 min), before the resin was washed thoroughly with DMF/DCM (1:1 v/v, 3 × 2.0 mL), DMF/DIPEA (95:5 v/v, 3 × 2.0 mL) and DMF (3 × 2.0 mL). Coupling was performed as double coupling at 65 °C (2 × 20 W, 20 min). For each coupling reaction, the required Boc-D-β-amino acid (3.0 eq), HOAt (3.0 eq) and HATU (2.8 eq) were dissolved in 500 µL 0.8 M LiCl in DMF/DMSO (4:1 v/v). For Boc-D-nucleo-β³-amino acids, fewer equivalents were used (2.0 eq Boc-D-β-amino acid, 2.0 eq HOAt and 1.9 eq HATU). Because of low solubility these building blocks were dissolved by sonification. DIPEA and 2,6-lutidine were added to the activated amino acid building block to yield an end concentration of 200 mM and 300 mM respectively before addition to the resin. After the second coupling step, the resin was washed with NMP (5 × 5.0 mL). Eventually remaining free amino groups were capped using DMF/DIPEA/Ac_2O (8:1:1 v/v, 1 × 2.0 mL, 5 min) before the resin was washed again with DMF/DCM (1:1 v/v, 3 × 2.0 mL), DMF/DIPEA (95:5 v/v, 3 × 2.0 mL) and DMF (3 × 2.0 mL). After completion of the peptide sequence, the resin was thoroughly washed with DMF/DCM (1:1 v/v, 3 × 2.0 mL), DMF/DIPEA (95:5 v/v, 3 × 2.0 mL), DMF (3 × 2.0 mL) and MeOH (3 × 2.0 mL).

Before labeling or acetylation of the β-peptides, the N-terminal lysine side chain was Fmoc-deprotected by addition of 20 % piperidine in DMF (v/v, 2 × 2.0 mL, 10 min) followed by thorough washing with DMF/DCM (1:1 v/v, 3 × 2.0 mL), DMF/DIPEA (95:5 v/v, 3 × 2.0 mL) and DMF (3 × 2.0 mL). The β-peptides **1**, **3**, **5**, **7**, **9**, **11**, **13**, and **15** were then acetylated with DMF/DIPEA/Ac_2O (8:1:1 v/v, 1 × 2.0 mL, 5 min). For NBD-labeling of the β-peptides **4**, **8**, **12**, **16**, **17** and **18**, 7-nitrobenz-2-oxa-1,3-diazol-4-yl (3.0 eq) and DIPEA (14.0 eq) dissolved in 500 µL DMF were added to the resin and incubated at rt overnight. For TAMRA-labeling of the β-peptides **2**, **6**, **10** and **14**, 5(6)-carboxytetramethylrhodamine (3.0 eq), PyBoP (2.8 eq) and DIPEA (6.0 eq) dissolved in 500 µL DMF were added to the resin and incubated at rt overnight. Afterwards, the resin of all β-peptides was washed with DMF/DCM (1:1 v/v, 3 × 2.0 mL), DMF/DIPEA (95:5 v/v, 3 × 2.0 mL), DMF (3 × 2.0 mL) and MeOH (3 × 2.0 mL).

Cleavage of the β-peptides was performed by addition of the cleavage mixture of TFA/*m*-cresol/tioanisole/EDT/TFMSA (10:1:1:0.5:1 v/v) to the resin. First the cleavage solution was added without TFMSA and incubated for 5 min on ice before TFMSA was added drop-wise to the icecold mixture. After incubation for 1 h on ice and 2 at room temperature on a shaker, the cleavage solution was filtered from the resin and collected. After concentrating the solution in a nitrogen stream, the crude β-peptide was precipitated with ice-cold diethyl ether and centrifuged for 20 min at -15 °C with 9000 rpm.

The supernatant was removed and the pellet was washed three times with ice-cold ether and dried. Afterwards, the crude β-peptides were purified via HPLC.

Analytical Data of the β-Peptides

Peptide 1
HPLC (Semi-prep., gradient: 20–40 % B in 30 min, λ in nm: 215, 245, 280): t_R = 9.3 min **ESI-MS** (m/z): 482.1 $[M+5H]^{5+}$, 602.4 $[M+4H]^{4+}$, 802.8 $[M+3H]^{3+}$. **HR-MS** (ESI): calc. for [C115H197N32O24] ($[M+5H]^{5+}$): 482.1030, found: 482.1028; calc. for [C115H196N32O24] ($[M+4H]^{4+}$): 602.3770, found: 602.3770; calc. for [C115H195N32O24] ($[M+3H]^{3+}$): 802.8335, found: 802.8329.

Peptide 2
HPLC (Semi-prep., gradient: 20–30 % B in 30 min, λ in nm: 215, 245, 540): t_{R1} = 23.0 min, t_{R2} = 25.4 min **ESI-MS** (m/z): 463.6 $[M+6H]^{6+}$, 556.1 $[M+5H]^{5+}$, 694.9 $[M+4H]^{4+}$, 926.2 $[M+3H]^{3+}$. **HR-MS** (ESI): calc. for [C138H216N34O27] ($[M+6H]^{6+}$): 463.6090, found: 463.6087; calc. for [C138H215N34O27] ($[M+5H]^{5+}$): 556.1294, found: 556.1289; calc. for [C138H214N34O27] ($[M+4H]^{4+}$): 694.9099, found: 694.9092; calc. for [C138 H213 N34 O27] ($[M+3H]^{3+}$): 926.2108, found: 926.2093.

Peptide 3
HPLC (Semi-prep., gradient: 20–30 % B in 30 min, λ in nm: 215, 245, 280): t_R = 9.3 min **ESI-MS** (m/z): 483.9 $[M+5H]^{5+}$, 604.6 $[M+4H]^{4+}$, 805.8 $[M+3H]^{3+}$. **HR-MS** (ESI): calc. for [C115H196N35O22] ($[M+5H]^{5+}$): 483.9053, found: 483.9055; calc. for [C115H195N35O22] ($[M+4H]^{4+}$): 604.6298, found: 604.6301; calc. for [C115H194N35O22] ($[M+3H]^{3+}$): 805.8374, found: 805.8373.

Peptide 4
HPLC (Semi-prep., gradient: 20–30 % B in 30 min, λ in nm: 215, 245, 464): t_R = 22.4 min **ESI-MS** (m/z): 508.1 $[M+5H]^{5+}$, 634.9 $[M+4H]^{4+}$, 846.2 $[M+3H]^{3+}$. **HR-MS** (ESI): calc. for [C119H195N38O24] ($[M+5H]^{5+}$): 508.1036, found: 508.1038; calc. for [C119H194N38O24] ($[M+4H]^{4+}$): 634.8777, found: 634.8781; calc. for [C119H193N38O24] ($[M+3H]^{3+}$): 846.1678, found: 846.1679.

Peptide 5
HPLC (Semi-prep., gradient: 15–25 % B in 30 min, λ in nm: 215, 245, 280): t_R = 24.4 min **ESI-MS** (m/z): 487.1 $[M+5H]^{5+}$, 608.6 $[M+4H]^{4+}$, 811.2 $[M+3H]^{3+}$, 1216.2 $[M+2H]^{2+}$. **HR-MS** (ESI): calc. for [C115H196N35O23] ($[M+5H]^{5+}$): 487.1043, found: 487.1038; calc. for [C115H195N35O23] ($[M+4H]^{4+}$): 608.6286, found 608.6289; calc. for [C115H194N35O23] ($[M+3H]^{3+}$): 811.1690, found: 811.1695; calc. for [C115 H193 N35 O23] ($[M+2H]^{2+}$): 1216.2499, found: 1216.2485.

Peptide 6
HPLC (Semi-prep., gradient: 20–30 % B in 30 min, λ in nm: 215, 245, 540): t_{R1} = 23.4 min, t_{R2} = 25.6 min **ESI-MS** (m/z): 467.8 $[M+6H]^{6+}$, 561.1 $[M+5H]^{5+}$, 701.2 $[M+4H]^{4+}$. **HR-MS** (ESI): calc. for [C138H215N37O26] ($[M+6H]^{6+}$): 467.7768, found: 467.7766; calc. for [C138H214N37O26] ($[M+5H]^{5+}$): 561.1307, found: 561.1338; calc. for [C138H213N37O26] ($[M+4H]^{4+}$): 701.1615, found: 701.1612.

Peptide 7
HPLC (Semi-prep., gradient: 15–25 % B in 30 min λ in nm: 215, 245, 280): t_R = 19.2 min **ESI-MS** (m/z): 479.3 $[M+5H]^{5+}$, 598.6 $[M+4H]^{4+}$, 797.8 $[M+3H]^{3+}$, 1196.2 $[M+2H]^{2+}$. **HR-MS** (ESI): calc. for [C114H196N33O23] ($[M+5H]^{5+}$): 479.1031, found: 479.1031; calc. for [C114H195N33O23] ($[M+4H]^{4+}$): 598.6270, found: 598.6268; calc. for [C114H194N33O23] ($[M+3H]^{3+}$): 797.8336, found: 797.8332; calc. for [C114 H193 N33 O23] ($[M+2H]^{2+}$): 1196.2468, found: 1196.2458.

Peptide 8
HPLC (Semi-prep., gradient: 20–30 % B in 30 min, λ in nm: 215, 245, 464): t_R = 18.9 min **ESI-MS** (m/z): 503.3 $[M+5H]^{5+}$, 628.9 $[M+4H]^{4+}$, 838.2 $[M+3H]^{3+}$, 1256.7 $[M+2H]^{2+}$. **HR-MS** (ESI): calc. for [C118H195N36O25] ($[M+5H]^{5+}$): 503.3013, found: 503.3023; calc. for [C118H194N36O25] ($[M+4H]^{4+}$): 628.8748, found: 628.8763; calc. for [C118H193N36O25] ($[M+3H]^{3+}$): 838.1640, found: 838.1659; calc. for [C118 H192 N36 O25] ($[M+2H]^{2+}$): 1256.7424, found: 1256.7414.

Peptide 9
HPLC (Semi-prep., gradient: 15–25% B in 30 min, λ in nm: 215, 245, 280): t_R = 24.8 min **ESI-MS** (m/z): 482.3 $[M+5H]^{5+}$, 602.6 $[M+4H]^{4+}$, 803.2 $[M+3H]^{3+}$, 1204.2 $[M+2H]^{2+}$. **HR-MS** (ESI): calc. for [C114H196N33O24] ($[M+5H]^{5+}$): 482.3021, found: 482.3008; calc. for [C114H195N33O24] ($[M+4H]^{4+}$):

602.6258, found: 602.6258; calc. for [C114H194N33O24] ($[M+3H]^{3+}$): 803.1653, found: 803.1655; calc. for [C114 H193 N33 O24] ($[M+2H]^{2+}$): 1204.2443, found: 1204.2455.

Peptide 10
HPLC (Semi-prep., gradient: 20–30 % B in 30 min, λ in nm: 215, 245, 540): t_{R1} = 22.4 min, t_{R2} = 24.8 min **ESI-MS** (m/z): 463.8 $[M+6H]^{6+}$, 556.3 $[M+5H]^{5+}$, 695.2 $[M+4H]^{4+}$, 926.5 $[M+3H]^{3+}$. **HR-MS** (ESI): calc. for [C137H215N35O27] ($[M+6H]^{6+}$): 463.7749, found: 463.7761; calc. for [C137H214N35O27] ($[M+5H]^{5+}$): 556.3284, found: 556.3294; calc. for [C137H213N35O27] ($[M+4H]^{4+}$): 695.1587, found: 695.1591; calc. for [C137 H212 N35 O27] ($[M+3H]^{3+}$): 926.5425, found: 926.5433.

Peptide 11
HPLC (Semi-prep., gradient: 15–25 % B in 30 min, λ in nm: 215, 245, 280): t_R = 23.8 min **ESI-MS** (m/z): 484.1 $[M+5H]^{5+,}$ 604.9 $[M+4H]^{4+}$, 806.2 $[M+3H]^{3+}$, 1208.8 $[M+2H]^{2+}$. **HR-MS** (ESI): calc. for [C114H195N36O22] ($[M+5H]^{5+}$): 484.1044, found: 484.1047; calc. for [C114H194N36O22] ($[M+4H]^{4+}$): 604.8787, found: 604.8792; calc. for [C114H193N36O22] ($[M+3H]^{3+}$): 806.1692, found: 806.1698; calc. for [C114 H192 N36 O22] ($[M+2H]^{2+}$): 1208.7500, found: 1208.7516.

Peptide 12
HPLC (Semi-prep., gradient: 20–30 % B in 30 min, λ in nm: 215, 245, 464): t_R = 18.8 min **ESI-MS** (m/z): 508.3 $[M+5H]^{5+}$, 635.1 $[M+4H]^{4+}$. **HR-MS** (ESI): calc. for [C118H194N39O24] ($[M+5H]^{5+}$): 508.3026, found: 508.3024; calc. for [C118H193N39O24] ($[M+4H]^{4+}$): 635.1265, found: 635.1261.

Peptide 13
HPLC (Semi-prep., gradient: 30–40 % B in 30 min, λ in nm: 215, 245, 280): t_R = 11.8 min **ESI-MS** (m/z): 515.9 $[M+5H]^{5+}$, 644.7 $[M+4H]^{4+}$, 859.2 $[M+3H]^{3+}$. **HR-MS** (ESI): calc. for [C126H220N33O24] ($[M+5H]^{5+}$): 515.9396, found: 515.9396; calc. for [C126H219N33O24] ($[M+4H]^{4+}$): 644.6727, found: 644.6728; calc. for [C126H218N33O24] ($[M+3H]^{3+}$): 859.2279, found: 859.2278.

Peptide 14
HPLC (Semi-prep., gradient: 30–40 % B in 30 min, λ in nm: 215, 245, 540): t_{R1} = 17.8 min, t_{R2} = 20.7 min. **ESI-MS** (m/z): 421.7 [M+7H]7+, 491.8 [M+6H]6+, 590.0 [M+5H]5+, 7737.2 [M+4H]4+, 982.6 [M+3H]3+. **HR-MS** (ESI): calc. for [C149H240N35O27] ([M+7H]7+): 421.6921, found: 421.6911; calc. for [C149H239N35O27] ([M+6H]6+): 491.8062, found: 491.8062; calc. for [C149H238N35O27] ([M+5H]5+): 589.9660, found: 589.9660; calc. for [C149 H237 N35 O27] ([M+4H]4+): 737.2057, found: 737.2060; calc. for [C149 H236 N35 O27] ([M+3H]3+): 982.6051, found: 982.6055.

Peptide 15
HPLC (Semi-prep., gradient: 30–40 % B in 30 min, λ in nm: 215, 245, 280): t_R = 12.3 min **ESI-MS** (m/z): 517.7 $[M+5H]^{5+}$, 646.9 $[M+4H]^{4+}$, 862.2 $[M+3H]^{3+}$. **HR-MS** (ESI): calc. for [C126H219N36O22] ($[M+5H]^{5+}$): 517.7419, found: 517.7423; calc. for [C126H218N36O22] ($[M+4H]^{4+}$): 646.9256, found: 646.9261; calc. for [C126H217N36O22] ($[M+3H]^{3+}$): 862.2317, found: 862.2325.

Peptide 16
HPLC (Semi-prep., gradient: 30–40 % B in 30 min, λ in nm: 215, 245, 464): t_R = 20.2 min **ESI-MS** (m/z): 541.9 $[M+5H]^{5+}$, 677.2 $[M+4H]^{4+}$, 902.6 $[M+3H]^{3+}$, 1353.3 $[M+2H]^{2+}$. **HR-MS** (ESI): calc. for [C130H218N39O24] ($[M+5H]^{5+}$): 541.9402, found: 541.9406; calc. for [C130H217N39O24] ($[M+4H]^{4+}$): 677.1734, found: 677.1738; calc. for [C130H216N39O24] ($[M+3H]^{3+}$): 902.5621, found: 902.5628; calc. for [C130 H215 N39 O24] ($[M+2H]^{2+}$): 1353.3396, found: 1353.3406.

Peptide 17
HPLC (Semi-prep., gradient: 20–40 % B in 30 min, λ in nm: 215, 245, 464): t_R = 12.7 min **ESI-MS** (m/z): 526.8 $[M+4H]^{4+}$, 702.1 $[M+3H]^{3+}$, 1052.7 $[M+2H]^{2+}$. **HR-MS** (ESI): calc. for [C101H178N26O22] ($[M+4H]^{4+}$): 526.8397, found: 526.8390; calc. for [C101H177N26O22] ($[M+3H]^{3+}$): 702.1171, found: 702.1188; calc. for [C101H176N26O22] ($[M+2H]^{2+}$): 1052.6721, found: 1052.6729.

Peptide 18
HPLC (Semi-prep., gradient: 30–40 % B in 30 min, λ in nm: 215, 245, 464): t_R = 23.9 min **ESI-MS** (m/z): 455.3 $[M+5H]^{5+}$, 568.9 $[M+4H]^{4+}$, 758.5 $[M+3H]^{3+}$, 1136.8 $[M+2H]^{2+}$. **HR-MS** (ESI): calc. for [C113H203N26O22] ($[M+5H]^{5+}$): 455.3108, found: 455.3013; calc. for [C113H202N26O22] ($[M+4H]^{4+}$): 568.8866, found: 568.8869; calc. for [C113H200N26O22] ($[M+2H]^{2+}$): 1136.7660, found: 1136.7662.

CD Spectra

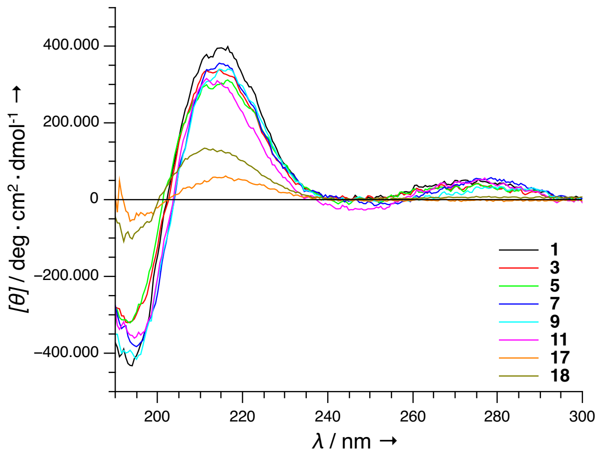

Figure S1. CD spectra of the indicated β-peptides at 20 °C in 10 mM Tris-HCl puffer at pH 7.5.

Concentration-dependent Fluorescence Spectra of the β-Peptides

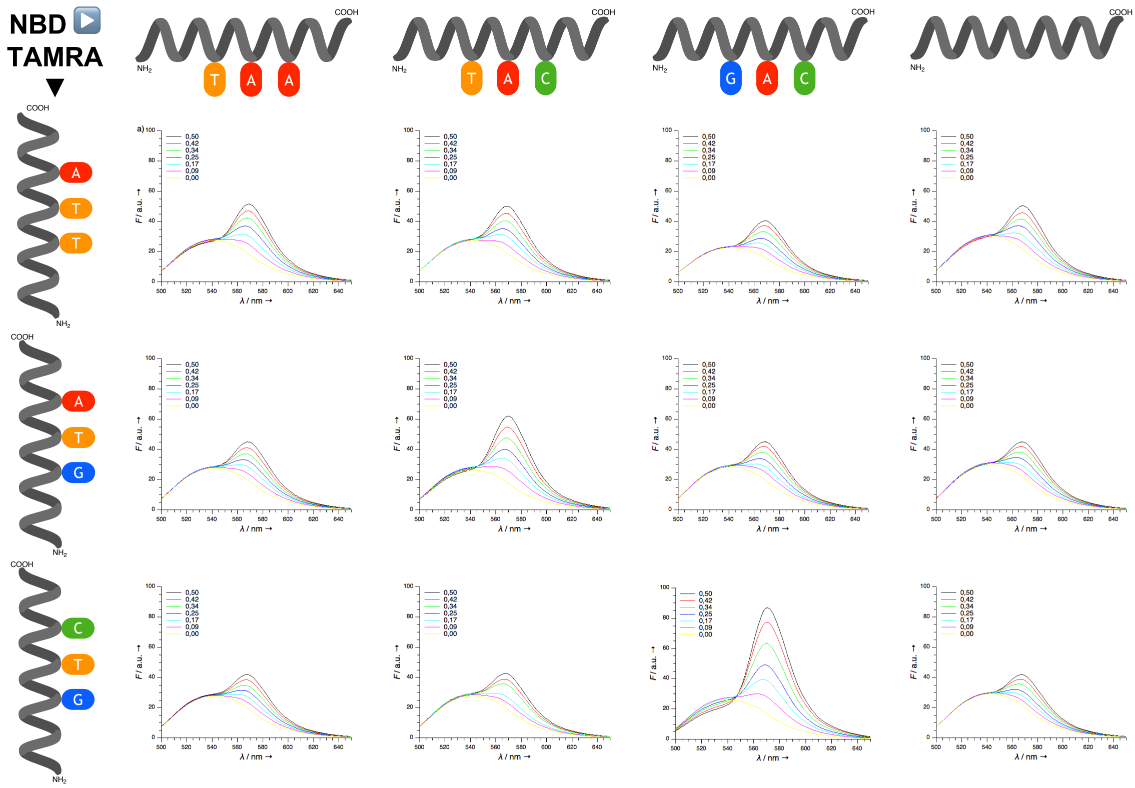

Figure S2. Fluorescence emission spectra of NBD-labeled β-PNA (4 µM) with the indicated molar fractions of TAMRA-labeled β-PNA (X_A = 0–0.5) and a total β-peptide concentration of 8 µM. Measurements were performed in 10 mM Tris-HCl buffer at pH 7.5 and at 10 °C.

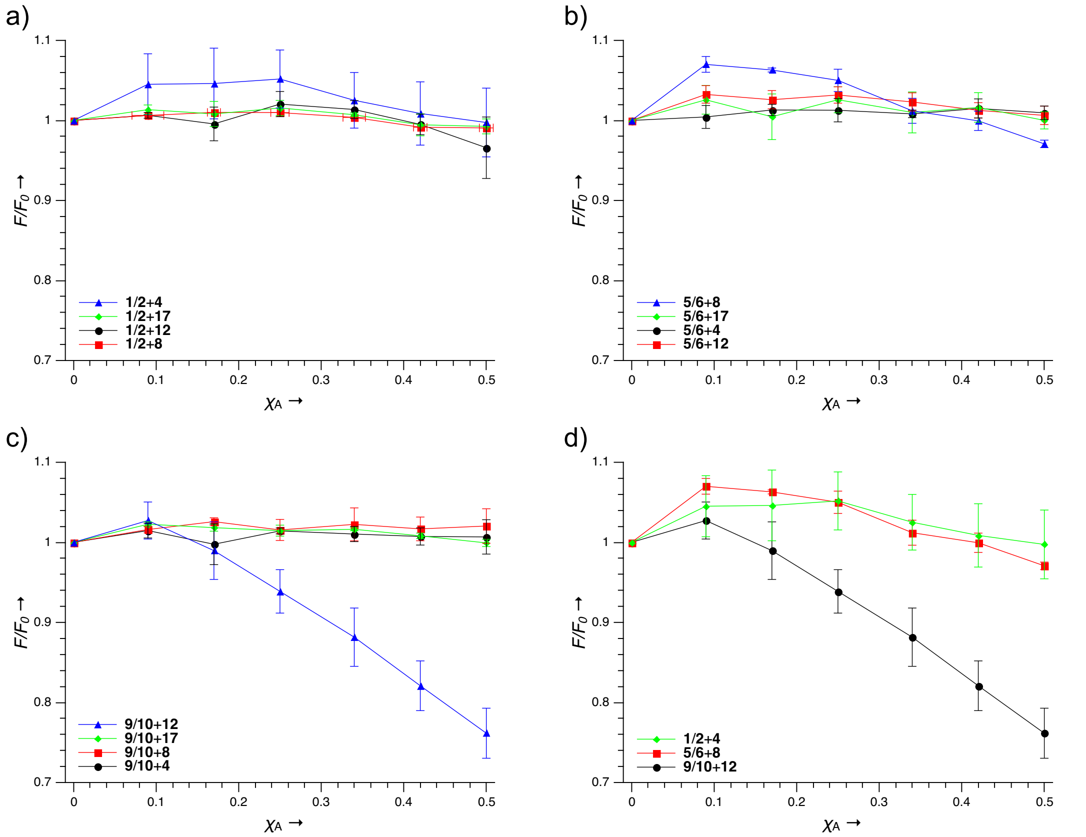

Figure S3. Relative changes in NBD fluorescence intensity (F/F_0) at 530 nm as a function of increasing molar fraction of the complementary TAMRA-labeled β-PNA strands (X_A). Shown are all possible combinations with **1/2** (a), **5/6** (b) and **9/10** (c) as well as the results of all matching combinations (d). Measurements were performed at 10 °C in 10 mM Tris-HCl buffer at pH 7.5 (n=3).

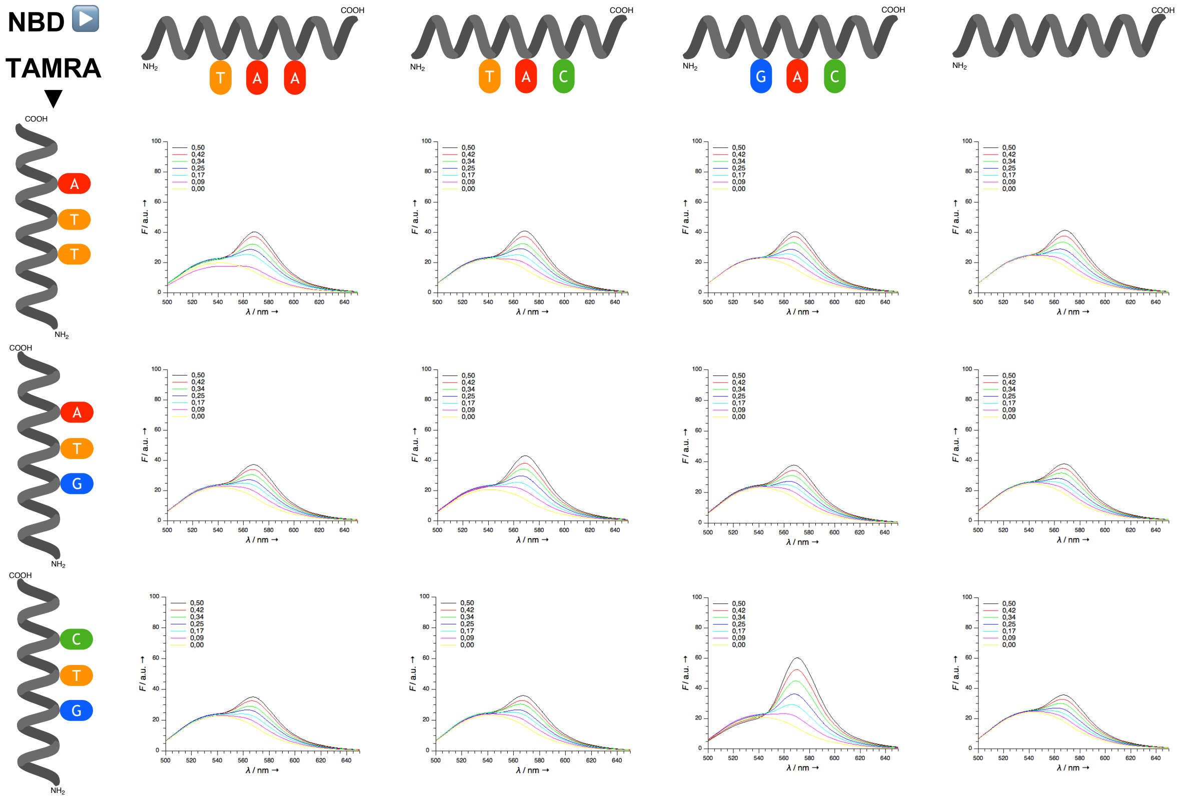

Figure S4. Fluorescence emission spectra of NBD-labeled β-PNA (4 µM) with the indicated molar fractions of TAMRA-labeled β-PNA (X_A = 0–0.5) and a total β-peptide concentration of 8 µM. Measurements were performed in 10 mM Tris-HCl buffer at pH 7.5 and at 20 °C.

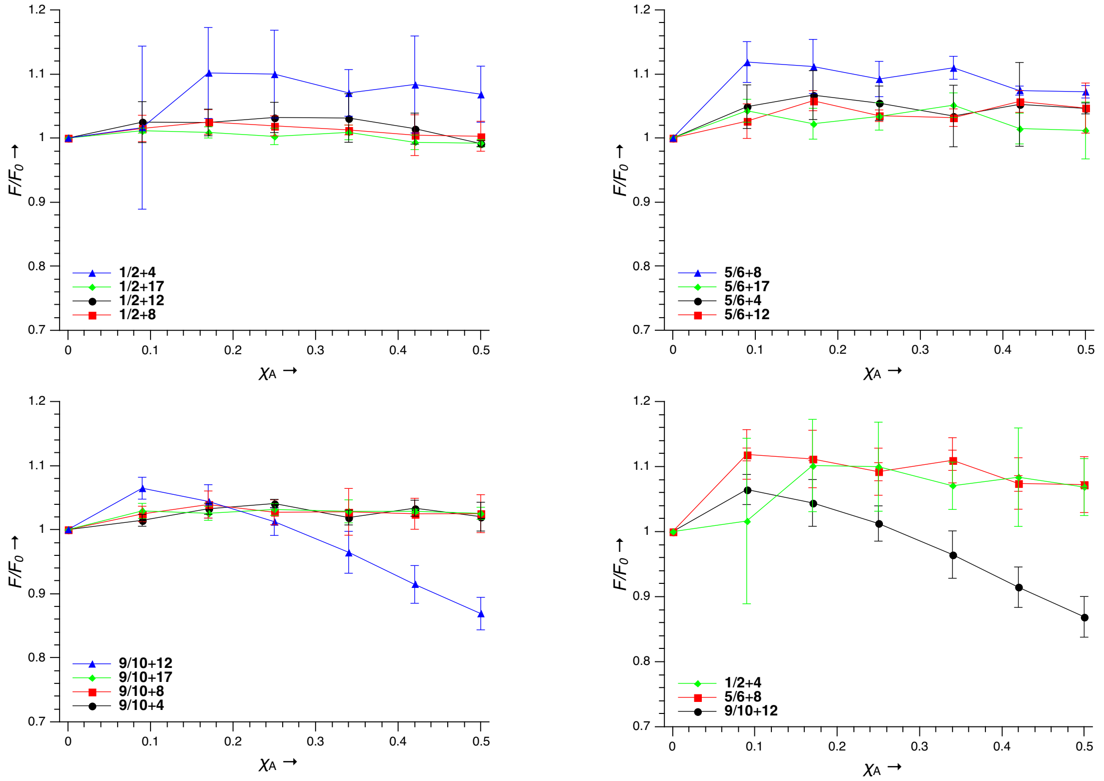

Figure S5. Relative changes in NBD fluorescence intensity (F/F_0) at 530 nm as a function of increasing molar fraction of the complementary TAMRA-labeled β-PNA strands (X_A). Shown are all possible combinations with **1/2** (a), **5/6** (b) and **9/10** (c) as well as the results of all matching combinations (d). Measurements were performed at 20 °C in 10 mM Tris-HCl buffer at pH 7.5 (n=3).

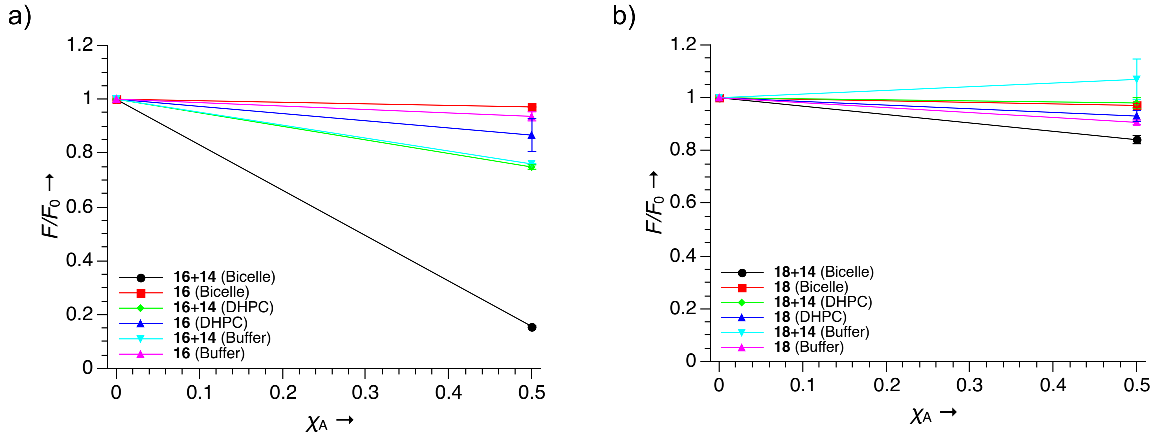

Figure S5a. Relative changes in NBD fluorescence intensity (F/F_0) at 530 nm as a function of increasing molar fraction of the complementary TAMRA-labeled β-PNA strands (X_A). Shown are measurements of **16** in presence of DMPC/DHPC bicelles (q=2), with DHPC or in buffer with and without equimolar addition of **14** (a) as well as of **18** under the same conditions as for **16** (b). Measurements were performed at 20 °C in 5 mM Tris-HCl buffer at pH 7.5 (n=3).

Temperature-dependent CD Spectra of the β-Peptides

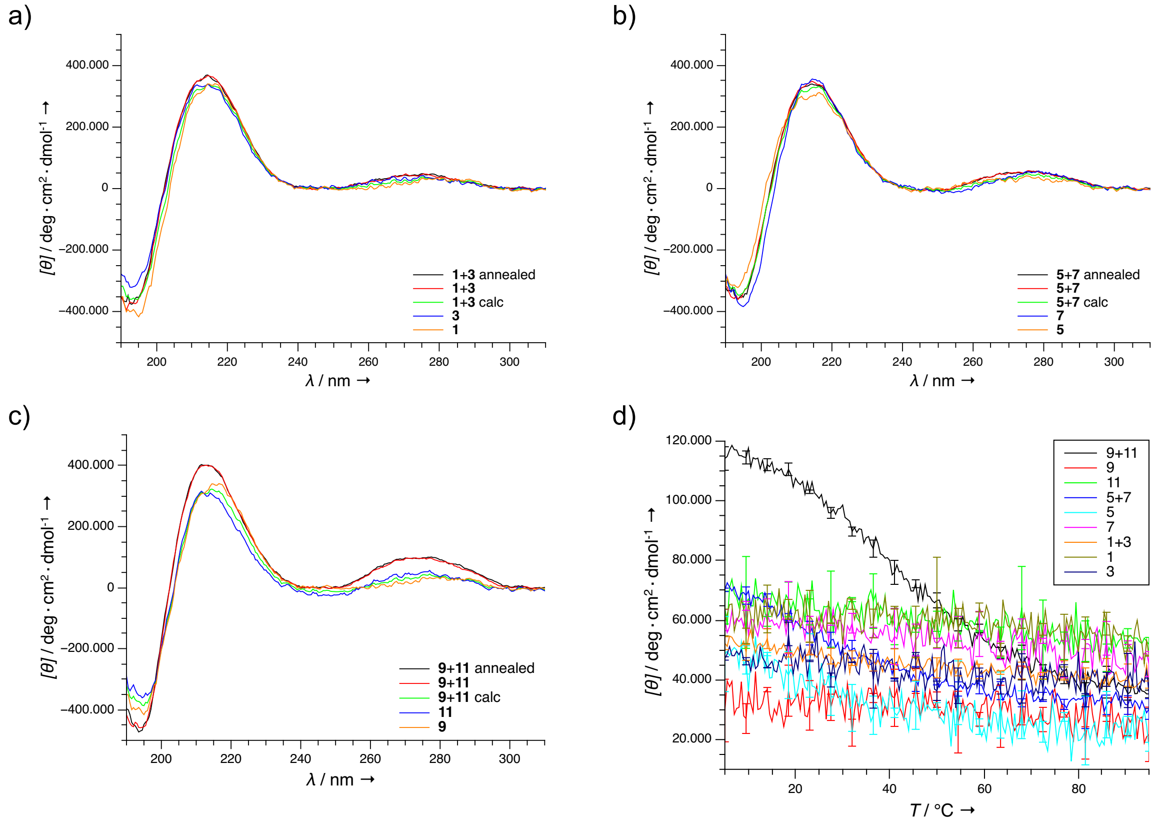

Figure S6. CD spectra of **1** and **3** (a), **5** and **7** (b) as well as **9** and **11** (c) measured at 20 °C separately, their calculated average and measured together with and without annealing at 80 °C. Temperature dependence of the CD spectra at 273 nm of the combinations **9+11**, **5**+**7** and **1+3** measured together and separately (d). Measurements were performed in 10 mM Tris-HCl puffer at pH 7.5 (*n*=3).

FRET Assay between β-Peptides 17, 18 and DOPC-LUVs

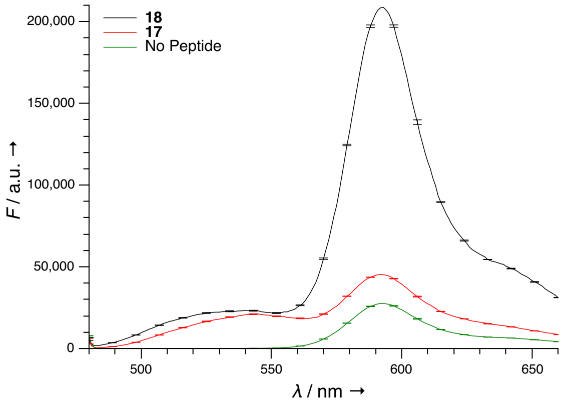

Figure S7. Fluorescence emission spectra of lissamin rhodamine B labeled DOPC-LUV in presence of **17**, **18** (5 µM) resulting in a P/L ratio of 1:150 or without β-peptides. Measurements were performed in 10 mM Tris-HCl buffer at pH 7.5 and rt (*n*=3).

FRET Assay between β-Peptides 16, 18 and 14 on Lipid Bilayers

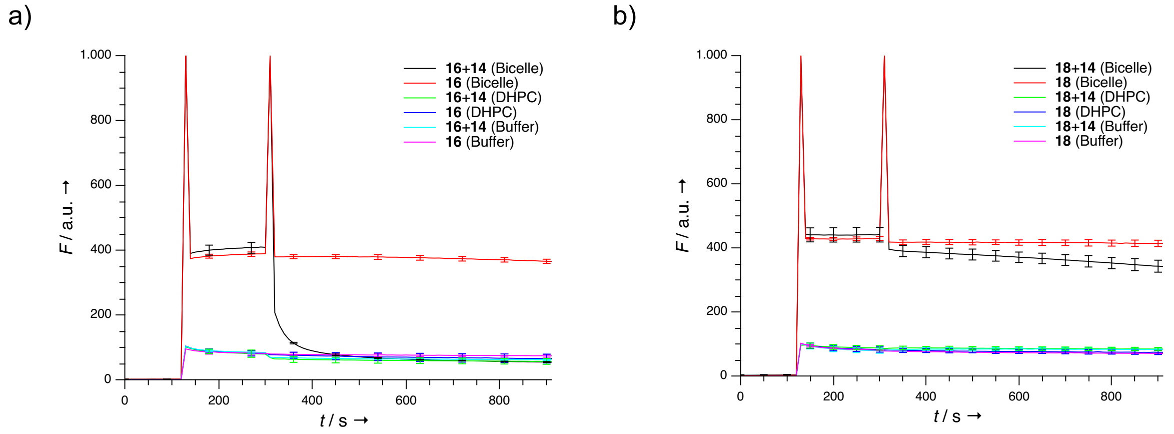

Figure S8. Time-resolved NBD fluorescence emission measurements of **16** in presence of DMPC/DHPC bicelles (q=2), with DHPC or in buffer with and without equimolar addition of **14** (a) as well as time-resolved NBD fluorescence emission measurements of **18** under the same conditions as for **16** (b). Measurements were performed at 20 °C in 5 mM Tris-HCl buffer at pH 7.5 (*n*=3).

CD Spectra of the β-Peptide Interaction on Lipid Bilayers

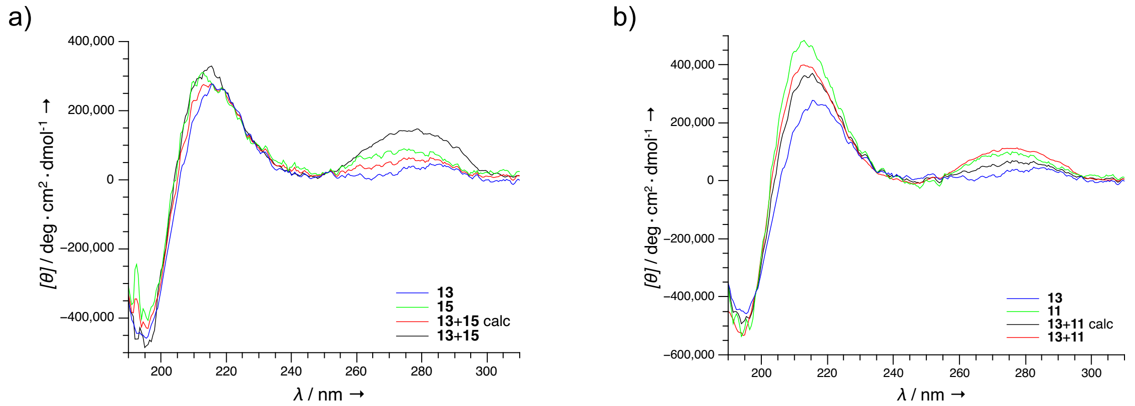

Figure S9. CD spectra of β-PNA interaction on the surface of DMPC/DHPC bilayers (q=2). **13** measured either together with **15** additionally with separate measurements and calculated average (a) or with **11** additionally with separate measurements and calculated average (b). Measurements were performed at 20 °C in 5 mM Tris-HCl buffer at pH 7.5 (n=3).

Characterization of LUVs and bicelles by DLS

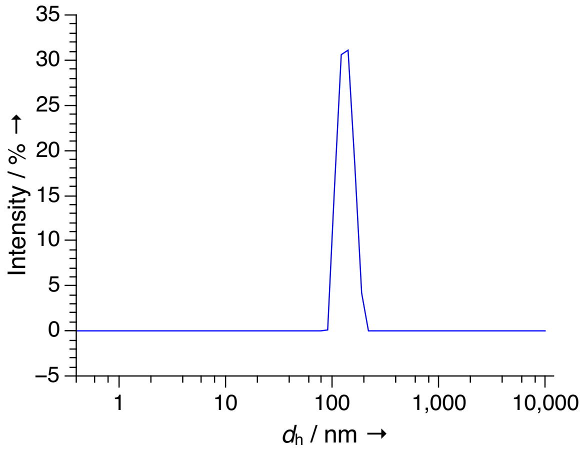

Figure S10. Mean size distribution plot determined by DLS of an LUV suspension with a mean hydrodynamic diameter of 133.8 nm prepared by extrusion through a polycarbonate membrane with 100~nm pore size. Measurements were performed at 25 °C in 10 mM TRIS-HCl buffer (pH 7.5).

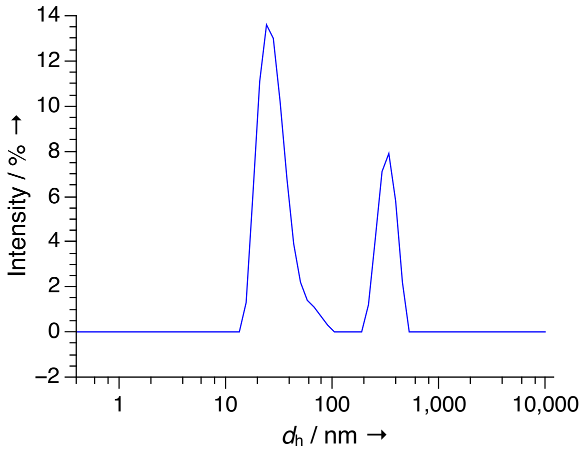

Figure S11. Mean size distribution plot determined by DLS of a bicelle suspension with a mean hydrodynamic diameter of 30.35 nm and 332.2 nm for the different size populations prepared from DMPC and DHPC with a q of 2. Measurements were performed at 25 °C in 10 mM TRIS-HCl buffer (pH 7.5).

References

[1] N. J. Greenfield, *Nat. Protoc.* **2007**, *1*, 2876–2890.
[2] R. C. MacDonald, R. I. MacDonald, B. P. M. Menco, K. Takeshita, N. K. Subbarao, L. Hu, *Biochim. Biophys. Acta - Biomembr.* **1991**, *1061*, 297–303.
[3] A. A. De Angelis, S. J. Opella, *Nat. Protoc.* **2007**, *2*, 2332–2338.
[4] P. Chakraborty, U. Diederichsen, *Chem. - Eur. J.* **2005**, *11*, 3207–3216.
[5] D. Seebach, M. Overhand, F. N. M. Kühnle, B. Martinoni, L. Oberer, U. Hommel, H. Widmer, *Helv. Chim. Acta* **1996**, *79*, 913–941.
